# Health Misinformation Detection in the Social Web: An Overview and a Data Science Approach

**DOI:** 10.3390/ijerph19042173

**Published:** 2022-02-15

**Authors:** Stefano Di Sotto, Marco Viviani

**Affiliations:** Department of Informatics, Systems, and Communication (DISCo), University of Milano-Bicocca, Edificio U14-ABACUS, Viale Sarca, 336, 20126 Milan, Italy; s.disotto@campus.unimib.it

**Keywords:** health misinformation, information disorder, consumer health, information access, social Web, machine learning, deep learning, data science

## Abstract

The increasing availability of online content these days raises several questions about effective access to information. In particular, the possibility for almost everyone to generate content with no traditional intermediary, if on the one hand led to a process of “information democratization”, on the other hand, has negatively affected the genuineness of the information disseminated. This issue is particularly relevant when accessing health information, which impacts both the individual and societal level. Often, laypersons do not have sufficient health literacy when faced with the decision to rely or not rely on this information, and expert users cannot cope with such a large amount of content. For these reasons, there is a need to develop automated solutions that can assist both experts and non-experts in discerning between genuine and non-genuine health information. To make a contribution in this area, in this paper we proceed to the study and analysis of distinct groups of features and machine learning techniques that can be effective to assess misinformation in online health-related content, whether in the form of Web pages or social media content. To this aim, and for evaluation purposes, we consider several publicly available datasets that have only recently been generated for the assessment of health misinformation under different perspectives.

## 1. Introduction

In contemporary society, access to information plays a crucial role, influencing choices and behaviors both at the level of individuals and communities. Web 2.0 technologies have enabled anyone to play an active role in every stage of the information life cycle, from its generation to its dissemination, especially through social media platforms. In this context, characterized by “disintermediation” [1,2], it is essential to be able to distinguish what is genuine information from what is not; this need is amplified, in particular, for those contents that may be particularly delicate and sensitive, because they could have extremely negative social repercussions, such as those related to health.

In this scenario, *health misinformation* can be defined as “a health-related claim of fact that is currently false due to a lack of scientific evidence” [3]. In most cases, people who are not an expert in the field are unable to properly assess the genuineness of such claims, both, in general, due to their limited cognitive capacities [4,5] and, more specifically, due to their insufficient level of *health literacy* [6]. This latter concept was included in the glossary of the *World Health Organization* (WHO) in 1988 [7], and indicates “the ability of a citizen to obtain, process, and understand basic health information in order to make informed choices”. Hence, the above-mentioned limitations, combined with the exponential increase in the amount of *user-generated content*, makes it necessary to develop methods that keep up with this growing trend and, at the same time, are able to reliably provide accurate predictions about the presence of health misinformation. This is because, in the vast majority of cases, laypersons are called upon to play an active role in managing their own health and that of others, contributing to social good [8].

Despite the recent technological and methodological improvements in addressing the problem of identifying distinct forms of non-genuine information—a.k.a. *information disorder* [9]—in several domains [10], the studies focusing on health misinformation detection are still quite limited. This is found both at the level of proposing domain-specific solutions, and in terms of the availability of appropriately labeled data with respect to the genuineness of such kind of information. In the last period, also concerning the COVID-19 pandemic and the general interest in access to health information online, this second problem has been somehow taken into account; in fact, some useful datasets for this purpose have been recently made available. This makes it possible, in this work, to study health misinformation features and machine learning solutions that can be employed for the problem under consideration, taking into account both Web and social media content from the Twitter microblogging platform.

Therefore, the main contributions of this work include, both from a theoretical and technological point of view, the following:

- Summarizing the characteristics of the Web, social media platforms, and health-related content being disseminated online, by considering factors of information genuineness in the health domain;
- Identifying key features (both “general-purpose” and domain-specific) that may be useful for detecting health misinformation in Web pages and social media content; this involves both synthesizing features that have been used in the literature for this purpose and investigating additional features that may be useful for the purpose considered, but employed to date for different research tasks;
- Studying the impact of such features when used in association with supervised learning techniques; this objective requires a comparison between different approaches that have been used so far to solve the problem considered, especially concerning “general” information (i.e., not health-related). This comparison involves the use of classical machine learning algorithms and the implementation of *Convolutional Neural Networks*, *Bidirectional Long Short-Term Memory* networks, and *Hierarchical Attention Networks*, which have been used in the literature to tackle document classification problems and are used in this work as an additional baseline for comparison;
- Evaluating the obtained results on publicly available datasets, which consider health misinformation in various communication media, in distinct forms, and with respect to various health-related topics.

## 2. An Overview on Health Misinformation Detection

Traditionally, the channel through which health-related content has been disseminated has always been an expert in the field, such as a physician or a recognized health organization, in an “extremely intermediate” way [11]. With the advent of modern communication channels such as newspapers, radio and TV, the intermediation aspect through experts has been somewhat loosened, as with time these channels have also begun to deal with this type of information. However, it is with the advent of the Web that the *expert–layperson relationship* has totally changed, providing anyone with a powerful means of getting health-related content without going through direct contact with the expert. In such a scenario, different solutions assessing health misinformation in Web pages and social media content have been proposed in the last few years.

Before introducing these solutions, it is necessary to underline that various concepts that are totally or partially superimposable to that of information genuineness (which, in general, we employ in this article) have been used. In the state-of-the-art works detailed in the next section, it is possible to find a reference, among others, to the concepts of *reliability*, *truthfulness*, *trustworthiness*, *credibility*, *veracity*, etc., which however can have specific meanings based on whether they refer to the source of information, the information itself, the communication medium through which information is propagated, or other theoretical aspects. Furthermore, reference was also made in the literature to the concepts of *disinformation*, understood as false information propagated with malicious intent, *misinformation*, understood as false information generated without malicious intentions, and *malinformation*, understood as genuine information shared to cause damage, often moving information destined to remain private in the public sphere [9]. In this article, as stated in the Introduction, we refer to the definition of health misinformation provided in [3], which makes no distinction between false information generated with the intent or not to cause harm.

### 2.1. Manual or Pseudo-Automated Approaches

In an attempt to understand the specific determinants *related* to online health misinformation, several studies were conducted starting from the early 2000s, especially in the fields of psychology and sociology, through the administration of *questionnaires* and by means of *survey analyses*. The main findings of these initial studies concerned aspects connected to the *trustworthiness* (and related concepts) of Web sites (in fact, social media was in its infancy) and the *credibility* (and related concepts) of the information published therein. They considered, among others, the Web site accessibility, the (perceived) quality of the content published, some of the users’ own characteristics, and the familiarity and habit in using the Web and content online [12]. The study presented in [13], in particular, reports and summarizes the following outcomes:

- The Web is characterized by the presence of a considerable amount of incomplete or poor-quality information, accompanied, however, by the presence of some excellent content and sources;
- Using keyword searches (*search by query*), instead of referring to known URLs (*navigational search*), increases the likelihood of stumbling upon less than credible health information;
- Most users make a hasty reading of content, without doing research related to the organization, author, or source of the health information being disseminated;
- When the source is taken into account, however, institutional sites are perceived as more credible than others whose source is unknown or not authoritative [14]. Furthermore, users tend to trust content produced, sponsored, or published by health care institutions and physicians (e.g., if the content link back to, or cite, professionals in the field). Conversely, “paid links” and broken links can reduce the credibility of a site or an article. However, there is no unequivocal agreement on a particular source, but it appears that differences in judgments are attributable to demographic factors and individual circumstances [15].

In particular, in [16], the relationship between *source expertise* and *knowledge of the domain* has been studied. The study found that in the case of unfamiliar content, the expertise of the source can somewhat influence users’ perceptions of the credibility of the information, while in the case of known content (or perceived as such), there is no significant interaction.

#### 2.1.1. Centralized Approaches

These are methods based on evaluations carried out by scientific authorities or expert organizations that are able to properly assess the information and thus ensure quality results. One of the main approaches that fall under this type of methodology is the one in which domain experts manually evaluate Web pages (and possibly social content) and, if some *quality criteria* are met, issue “badges” that prove the informational goodness of the content [17].

Alternatively, health-related content can be placed in a central collection, where users can be informed safely. This provides a knowledge base that can be employed for inferring new information, by modeling genuine information known by means of a *knowledge graph* against which the truthfulness of new information, possibly expressed in RDF triples, can be evaluated. In this way it would be a centralized and pseudo-automated method, as, for example, the one illustrated in [18] in the context of general misinformation detection. The advantage of such methods is that the results in terms of accuracy, quality, and, in general, genuineness of information are very high; however, relying on a manual process, they are particularly expensive and time-consuming. In addition, they do not scale with respect to the speed at which content grows and even how it is updated.

#### 2.1.2. Distributed Approaches

**Figure 1:**
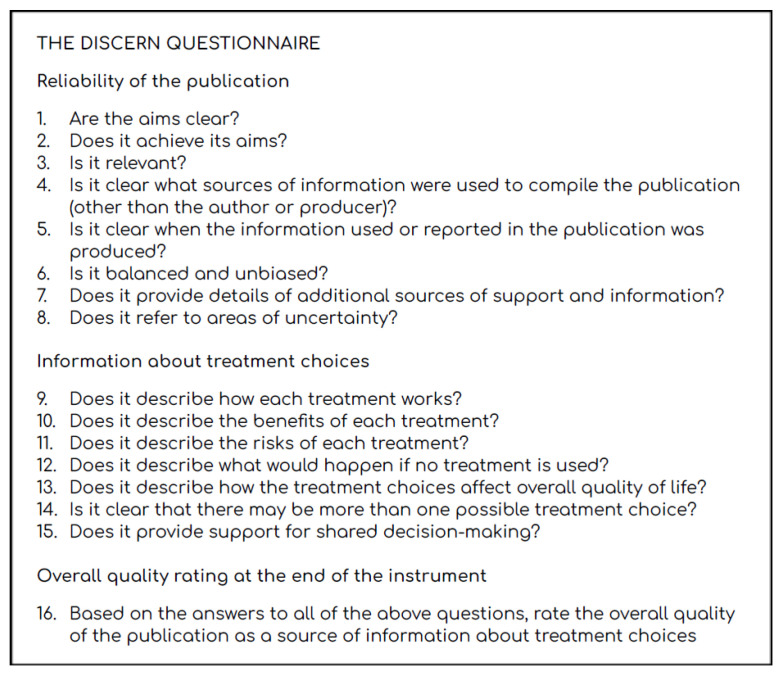
The DISCERN questionnaire. Graphic elaboration of the 16 questions extracted from http://www.discern.org.uk/discern_instrument.php (accessed on 3 February 2022).

These are methods based on the use of tools designed to allow any user to be able to distinguish information from misinformation even without prior medical knowledge. Among the various tools proposed is DISCERN [19], a system designed by the English national health system, whose accuracy has been tested on several occasions [20]. The advantage of using this approach is that it allows anyone to independently assess medical information regarding health treatments, based on “objective” criteria. These criteria are illustrated in Figure 1.

Disadvantages include the reluctance of users to carve out time to proceed with filling out the questionnaire, the fact that the process must be iterated over every piece of health-related content, and the low uptake of the method among the population. It should also be noted that this tool is designed for rather long texts that characterize Web pages; it has not been tested in the context of health misinformation detection in social media content.

### 2.2. Automated Approaches

These are methods that aim to assess the genuineness of health information automatically, without the direct intervention of users. A number of solutions have been proposed in recent years for misinformation detection in “general” online content for different domains and tasks, ranging from *opinion spam* to *fake news detection* [10,21,22,23]. However, only recently studies have been carried out in the health domain, also due to the growing impact of *Consumer Health Search* (CHS) [24,25].

The approach proposed in [17], considering Web content in the form of articles about health treatments, therapies, etc., aims to automate (a reduced version of) DISCERN. A *Hierarchical Encoder Attention-based* model is implemented, which exploits BERT [26] and BioBERT [27] to assign scores to Web pages with respect to the *completeness* and *accuracy* of five aspects form the abbreviated DISCERN. The model, despite its limitations related to the specificity of the medical information considered, obtains high performance, also by virtue of exploiting the semantics of the texts.

Another approach focusing on content analysis has been proposed in [28]. In a first method, two groups of medical Web pages are selected, i.e., *highly-credible* and *non-credible*. Through the TAGME application [29], the most representative topics are extracted from the two groups of pages. To evaluate the credibility of a new topic extracted from an unjudged Web page, the Jaccard similarity between the topic just extracted and those belonging to the two reference groups is computed. The method returns good values, but it has several limitations including dependence on the choice of the two reference groups. In a second proposed method a Markov model is trained using textual features, considered discriminating in identifying health misinformation. These include the use of business terms, certain specific punctuation elements, and certain parts of speech. The results obtained show that the identified features are suitable for modeling the problem at hand.

Recently, the graph-based DETERRENT model [30] has been proposed to detect misinformation in online health-related articles by leveraging on additional information from a *Medical Knowledge Graph* and on a propagation network built on such graph and on an *Article-Entity Bipartite Graph*. Empirical validation using two disease-specific datasets, i.e., related to diabetes and cancer, demonstrates the effectiveness of the model. Another recent solution, proposed in [8], aims at considering both the structure and the content of Web pages to perform a binary classification between health information and misinformation, by adding domain-specificity to the Web2Vec model developed for phishing Web page detection [31]. Features are extracted by means of a CNN-BiLSTM network; in particular, a Word2Vec layer is pre-trained on PubMed for content-based features, and an attention mechanism is applied to feature embedding representations. The results show, on three publicly available datasets, the effectiveness of considering health domain characteristics in the proposed approach.

One of the first studies explicitly addressing health misinformation in social media is the one proposed in [32]. The study presents a *Markov Random Field* (MRF) model, which jointly considers the *reliability of the user* (based on the *engagement* within the community and other information such as socio-demographic factors), of the provided *statements* (with regard to the medicines to be taken and the potential side effects, as reported in the *Mayo Clinic* dataset [33]), and their *linguistic objectivity* (identified based on the extraction of linguistic-stylistic but also linguistic-emotional features). The results obtained on 15,000 users and their 2,800,000 posts about the side effects of drugs and health products from *healthboards* [34] (one of the largest health-focused online communities) reach a good accuracy, even if, also in this case, the method is very disease-specific. Another disease-specific work examines social media users promoting cancer treatments that have been shown to be ineffective [35]. The authors study around 4000 Twitter users posting about such “treatments”, and compare them to a baseline of users generally interested in cancer, by considering features capturing user attributes, writing style, and sentiment. Such misinformation detection strategy presents an accuracy of over 90% for the considered disease.

In the last couple of years, some work based on the use of multiple features in conjunction with machine learning approaches has been proposed for health misinformation detection in social media. In [36], the authors describe (in addition to various baselines considered) an approach based on a traditional statistical learner such as SVM, in association with the *Linguistic Inquiry and Word Count* (LIWC) psycho-linguistic lexicon [37], from which 93 textual features are extracted. Other content-based approaches are illustrated in [36,38,39], where *Convolutional Neural Networks* (CNNs) are employed to classify health information with respect to its genuineness based on textual features. In the study presented in [40], the authors incorporate in supervised classification models *central-level features* (including topic features) and *peripheral-level features* (including linguistic, sentiment, and user behavioral features). The considered dataset (around 5000 items) has been collected from a real online health community focusing on autism. The results show that, in this case, behavioral features are more informative than linguistic features in detecting health misinformation. Another recent work aims to explore various features other than textual to identify health misinformation in social media [41]. Empirical data including around 1200 pieces of health information have been collected and manually labeled. The findings show that there are significant differences in the features of health misinformation between different topics.

In this article, as illustrated in the next section, we propose the use of supervised learning in association with a large number of different features, not all of which were considered together in previous works, for health misinformation detection. To do this, we test the effectiveness of such features on three publicly available datasets with a higher number of observations than those used so far in the literature, also to consider at the same time different types of content (both Web news/reviews and social media content) and different types of health-related topics, to avoid disease-specificity. Finally, we also consider other baselines that have been used in the literature for “general” misinformation detection. For example, *Hierarchical Propagation Networks* (HPNs) proposed in [42] for the identification of fake news, and *Bidirectional Long-Short Term Memory* (Bi-LSTM) networks employed for the identification of both fake news in [43] and rumors in [44], and also in the health domain for the identification of fake content related to COVID-19 [45].

## 3. Materials and Methods

This section initially presents the *datasets* that were considered in this paper and the *health misinformation features* extracted from such datasets, and then describes several *classifiers* used and tested to discern between information and misinformation.

### 3.1. Datasets

Following extensive research against resources available on the Web and in the literature, a low number of publicly available datasets containing health information labeled with respect to its genuineness were found. Among the best we have identified include the *CoAID* dataset [38], the *ReCOVery* dataset [36], and the *FakeHealth* dataset [39]. While *CoAID* and *ReCOVery* contain “informal” health-related content about the COVID-19 pandemic, *FakeHealth* contains expert-generated health information on various medical topics.

#### 3.1.1. CoAID

It is a collection of *news* and *claims* written in English, having the COVID-19 pandemic as the main subject of discussion. In detail, by “news” the authors mean classic articles about events and/or public-interest information, while by “claim” they mean short texts that are generally composed of a maximum of two sentences. Depending on the type of information considered, authors performed a different data collection flow.

- *News*: are considered as “credible” news those extracted from nine highly-reliable sources including public health institutions or authorities, such as: *Healthline* [46], *ScienceDaily* [47], the *National Institutes of Health* (NIH) [48], *MedicalNews Today* (MNT) [49], the *Mayo Clinic* [50], the *Cleveland Clinic* [51], WebMD [52], the *World Health Organization* (WHO) [53], and the *Centers for Disease Control and Prevention* (CDC) [54]. As for “not credible” news, the authors include those reported by various sites involved in *fact-checking* (e.g., WHO and MNT) as false myths.
- *Claims*: they are sourced from the official Web site and the official Twitter account of the WHO and from the MNT Web site. These claims are information expressed in the form of answers to common questions or false myths related to COVID-19.

For each piece of collected content, whether in the form of news or claims, the dataset is provided with the IDs of the *tweets* that the authors collected that refer to them. With respect to such tweets, the IDs of the corresponding *replies* are also provided.

#### 3.1.2. ReCOVery

It is a collection of *news items* written in English related to COVID-19, collected from around 60 different news Web sites. The “reliability” of news items is assessed by an automated approach that does not involve the use of domain experts. Specifically, it is derived based on the trustworthiness of its source. To assess such trustworthiness, both *NewsGuard* [55], and *Media Bias/Fact Check* (MBFC) [56], two popular fact-checking sites, are employed. Both sites produce reliability ratings: a news item is labeled as reliable if it exceeds certain thresholds for both sites, as unreliable otherwise. As in the cases of *CoAID*, also for *ReCOVery* the IDs of the *tweets* that refer to the news items taken into consideration are provided, but the IDs of the corresponding *retweets* and *replies* are not made available.

#### 3.1.3. FakeHealth

It is a collection of *reviews* generated by experts regarding health-related content about medical interventions, wellness, therapies, etc., written in English. Such reviews are published on the *HealthNewsReview* Web site [57], which is the Web interface of a project active from 2005 to 2018 and supported by the *Informed Medical Decisions Foundation* [58]. This project involved at least two independent reviewers with years of experience to evaluate, according to 10 criteria, the “reliability” of the more than 2500 total health-related contents analyzed. As for *CoAID*, also in *FakeHealth* the various contents are distinguished according to the type of source: we speak of *Story* if the source is a media, or of *Release* if the source is a health institution, a university, etc. The reliability assessment is performed by considering 10 different criteria detailed in [39], concerning for example the completeness of information about treatments, the presence of disease-mongering, the usage of independent sources, etc. Additionally, in this case, for each piece of collected content, the dataset is provided with the IDs of the *tweets* related to it. With respect to such tweets, the IDs of the corresponding *retweets* and *replies* are also provided.

#### 3.1.4. Data Gathering and Cleaning

Table 1 summarizes the numerosity of the data made available in each dataset, with respect to their nature.

To extract the features related to the propagation network (see Section 3.2.5) and user profiles (see Section 3.2.6), it was necessary to proceed with a further *data gathering* phase, by means of the Twitter API starting from the available tweet IDs. Hence, for each tweet ID, the following data has been gathered: *message text*, *date and time* of the message, *number of retweets*, *number of favorites*, *user* ID (i.e., the author of the tweet), and the associated *user profile description*, *number of followers*, and *number of followees*. The same data were gathered for *retweets* and *replies* associated with the considered tweet ID. Because the data in the *CoAID*, *ReCOVery*, and *FakeHealth* datasets were collected some time prior to this work, it occurred that not all of the original tweet IDs were still accessible during the data gathering phase. This is because, in the meantime, messages have been removed from the platform as not compliant with Twitter policies, some users have deleted their profiles, or have been suspended. It was thus possible to verify that the number of inaccessible tweet IDs is significant for each dataset. At a general level, *FakeHealth* (*Release*) and *FakeHealth* (*Story*) are those incurring in the lower reduction compared to *CoAID* and *ReCOVery*. Furthermore, the health-related content collected within the *FakeHealth* (*Story*) dataset is, among the considered datasets, the one that has generated more activity on Twitter.

Following the data gathering phase, a *data cleaning* phase was also carried out to eliminate duplicates and remove HTML codes and undocumented characters in the texts and links pointing to images, which are not considered in this work. The numerosity of the data used in this work following the data gathering and cleaning phases on the considered datasets is shown in Table 2.

Further details about the employed datasets, together with the code developed to gather and clean data, to extract the considered features, and to implement the proposed classifiers, as well as further documentation about the extracted features, are publicly accessible at the following link: https://github.com/ikr3-lab/health-misinformation/ (accessed on 3 February 2022).

### 3.2. Health Misinformation Features

Based on the literature and on a classification work performed in this article, we identify six classes of *health misinformation features*: (i) *textual representation* features, i.e., relating to different possible formal representations of the text, (ii) *linguistic-stylistic* features, i.e., taking into account the presence of different stylistic aspects of the text, (iii) *linguistic-emotional* features, i.e., identifying aspects of emotional character that transpire from the text, (iv) *linguistic-medical* features, i.e., related to the presence of specific medical terms within the text, (v) *propagation-network* features, i.e., taking into account the social network and the way information is propagated on it, and (vi) *user-profile* features, i.e., related to various metadata connected to user profiles.

#### 3.2.1. Textual Representation Features

Both the *Bag-of-Words* (BoW) and the *word embedding* representation models are employed to extract such features, which underlie a variety of approaches to detect misinformation, both general and health-related [10,32,59,60]. *Bag-of-Words features* are constituted by the unique terms that appear within the documents (i.e., health-related textual content). In the case under consideration, two different weighting schemes are considered: (i) *binary weighting*, where the presence or absence of the term within the document is indicated with a binary value associated with the term, and (ii) *Term Frequency—Inverse Document Frequency* (TF-IDF) *weighting*, where the frequency of a term within the document related to the number of occurrences of the term in the document collection is associated with the term. *Word embedding features* are constituted by real-valued vectors encoding the meaning of each word, such that words that are closest in vector space are expected to be similar in meaning [61]. In this work, the GloVe model has been used to perform embedding of words [62]. Specifically, the model was pre-trained on Wikipedia 2014 + English Gigaword Fifth Edition corpora [63], having sizes of 50, 100, and 200. The total number of features obtained by means of the above text representation models depends on the dataset under consideration. This information is, therefore, provided in the shared documentation.

#### 3.2.2. Linguistic-Stylistic Features

These features capture the set of stylistic traits that characterize a text. They have already been employed in assessing the credibility of online health information, such as in [28,32]. In the context of generic medical-themed Web pages, the study presented in [28] noticed that many sentences that provide credible information are expressed in the passive form. With reference to health-related social media content, in [32] the authors have associated constructs expressing *uncertainty* with non-credible information. Such constructs are identified in strong modals, possibility adverbs (e.g., maybe, probably, etc.), conditional and question particles (e.g., who, what, when, etc.). Conversely, credible information is associated with the use of constructs that provide some degree of certainty such as: weak modals, demonstrative adjectives/pronouns, and declarative conjunctions (e.g., therefore). Table 3 illustrates the list of the 25 features considered in this paper, extracted using *Part-of-Speech* (PoS) tagging by means of the NLTK toolkit [64].

#### 3.2.3. Linguistic-Emotional Features

These features concern the possible emotional aspect expressed by a text. An example of the usage of such features for health misinformation detection can be found in [32,40]. Such features capture the degree of *objectivity*/*subjectivity* of a text, its *polarity*, and the *emotions* it conveys. An objective text is one in which the information is expressed from a general point of view, is supported by objective data and there is no personal or emotional involvement of the author. In contrast, a subjective text is characterized by the expression of information through the author’s views, beliefs, and emotions. The polarity of a text, captured by *sentiment analysis* techniques [65], is the expression that determines the sentimental aspect of an opinion; in the case of an objective text, it is by definition neutral, while, in the presence of a subjective text, it can be positive or negative, with different levels of *intensity*. The presence of polarity, especially when the intensity is high, is likely to indicate bias and, therefore, health information of questionable credibility [32]. Similarly, the presence of particular emotions, which can be extracted using *emotion analysis* techniques [65], can be associated with health misinformation. For example, texts expressing emotions such as anxiety and uncertainty are a symptom of information that is not always credible, while texts with little emotional involvement seem to be more credible [32].

In this work, to compute the objectivity, subjectivity, and polarity features, we used the algorithm proposed in the *TextBlob* library [66], which is based on a general-purpose lexicon (in the absence of an established literature on medical lexicons used in sentiment analysis techniques and semantic approaches pre-trained on such lexicons). To extract emotions, we employed two distinct solutions. The first solution makes use of the *NRC Emoticon Lexicon* (NRC) proposed in [67], which allows identifying the percentage of terms in the text that express a given emotion among the following ones: *anger*, *fear*, *anticipation*, *trust*, *surprise*, *sadness*, *joy*, and *disgust*. The second solution is based on the *text2emotion* library [68], which provides, for each text, a score in the [0, 1] interval with respect to the intensity detected for each of the following emotions: *happiness*, *anger*, *sadness*, *surprise*, and *fear*. In total, for this feature class, we extracted 38 features that, again, are better detailed in the shared documentation.

#### 3.2.4. Linguistic-Medical Features

They aim to capture statistics related to the appearance of specific medical (or health-related) terms that impact health information genuineness. The 4 features considered for this category include: *normalized count of medical terms*, *normalized count of unique medical terms*, *hyperlink count*, and *normalized count of commercial terms*. The idea of using such features for this purpose was partly inspired by the outcomes illustrated in Section 2 with respect to the Web content scenario, in particular concerning the count of hyperlinks and commercial terms. Details about these features are provided below.

- *Normalized count of medical terms*: this is a count of the medical terms present in a given text normalized by the total number of words. Extracting this feature required the use of a *Named-Entity Recognition* (NER) model specially trained on medical information, namely the *spaCy* library [69]. Such a model is particularly suitable since it is trained on *MedMentions* [70], a collection of 4392 titles and abstracts published in *PubMed* [71], manually annotated by a team of experts. The main limitation of using this application lies in the fact that it is not able to recognize terms introduced in the medical-scientific language after the training operation (in the specific case that occurred in 2018). To overcome this, the output of the model has been supplemented with manual extraction of all terms related to COVID-19 and not present in the original dictionary. This list of words is the same as that used to generate the COVID-19 stream made available by Twitter for research purposes and accessible at the following link: https://developer.twitter.com/en/docs/labs/covid19-stream/overview/ (accessed on 3 February 2022).
- *Normalized count of unique medical terms*: in this case, the (normalized) unique count of medical terms is considered. The rationale behind this choice is because we expect that a high number of distinct medical terms corresponds to a higher mastery of the specific language and therefore of the specific medical domain.
- *Hyperlink count*: the presence of external hyperlinks can be associated with misinformation when such links point to misleading and/or advertising content. The computation of such feature was done by counting the number of *hyperlinks*, extracted using appropriate regular expressions.
- *Normalized count of commercial terms*: As illustrated in the literature [13], the higher the number of commercial terms, the less credible is perceived the related information, due to the for-profit purpose of such information. At a practical level, a list of 45 commercial terms taken from [72] (such as “*sale*”, “*deal*”, “*ad*”, etc.) has been compiled. The frequency of such terms in the health-related content has been computed and normalized on the basis of the total number of terms present in the considered text.

#### 3.2.5. Propagation-Network Features

Recent works have identified some characteristic patterns that differ between information propagation related to genuine (general) news and those related to fake (general) news [42]. Making explicit reference to Twitter (i.e., the social media platform on which some social media features have been extracted and evaluated, as later illustrated in Section 4), gives the possibility to reason on different kinds of relationships, even oriented. Specifically, so-called *macro interactions* are those made explicit by the “retweeted by” relationship, and *micro interactions* are represented by the “reply to” relationship. In this work, by referring to the features proposed in [42] and additional features, we considered the following four categories.

- *Structural features*: variables designed to capture aspects of network structure and topology. They refer to characteristics such as *depth*, *breadth*, and *out-degree* (in this case representing a measure of popularity) at both the global and cascade network levels;
- *Temporal features*: variables whose goal is to capture temporal aspects related to information dissemination. They refer to characteristics such as *duration of dissemination*, *average speed of dissemination*, and *average speed of response* at both the global network and cascade levels;
- *Linguistic features*: variables designed to capture the linguistic aspects of messages that interact with information dissemination. These variables are only applied within the micro-network, since at the *retweet* level, and to a large extent at the *tweet* level, the recorded messages are the same. This group of features relates to the sentiment analysis of the above texts both globally and at the cascade level. In this case, to assess the sentiment related to such short texts, we employed VADER [73], a lexicon and rule-based sentiment analysis tool that is particularly suitable for social media content;
- *Engagement features*: variables that assess the level of appreciation received by nodes expressed in the form of “favorites”.

Given their high numerosity, the 36 considered features belonging to the above four categories are described in detail in the documentation shared along with the data and code, at the link previously provided.

#### 3.2.6. User-Profile Features

This class of features (which may have been referred to by a different name in other works) have been widely used in assessing the genuineness of “general” information [10,21,23], and also proposed in recent works for health misinformation detection [40,41]. At a general level, information about user characteristics can be derived from any social media; in this paper, we refer to the Twitter platform. Therefore, the features considered are the *number of followers* and *followees*, the *number of medical terms in the profile description* of users, and the *active contribution* given by each user in the development of the network of information propagation. The expected behavior on the number of *followers* is that higher numbers correspond to higher authority and consequently more genuine information diffused. Instead, being a *followee* of many profiles can indicate not being a *bot* (bots often have many followers but follow few other accounts) [74]. User profile descriptions were analyzed to search within them for medical-scientific terms (again, extracted from PubMed, as in the case of linguistic-medical features). The hypothesis is that a greater number of such terms in the description corresponds to a greater knowledge of the health care domain and, therefore, more reliable information is expected. Regarding the individual contribution to the development of the propagation network, it is defined as *active contribution*, and can be measured in terms of *tweet posting*, *retweets*, and *replies* by each user in the virtual community. Excessive contribution by the individual user (especially in a short period of time) could be associated with a malicious behavior of a *bot*. Again, due to their rather high numerosity and specificity, the 16 user profile features considered as part of the proposed study are detailed in the shared documentation.

### 3.3. Health Misinformation Detection

In total, 119 different features were considered in this work, which have been used within *binary classifiers* to distinguish health information from misinformation. Some of them have been used in the literature in the context of both “general” and health misinformation detection, as illustrated in Section 2, and others have been employed for general-purpose document classification. In particular, a number of “classical” machine learning algorithms such as *Gradient Boosting*, *Logistic Regression*, *Naïve Bayes*, and *Random Forests* have been considered; furthermore, in order to exploit at best the features that rely on onerous and complex representation models such as *word embedding*, advanced algorithmic solutions have been implemented, such as those pertaining to the world of *Deep Learning* (DL), which are explained in detail in the following.

#### 3.3.1. Convolutional Neural Networks

**Figure 2:**
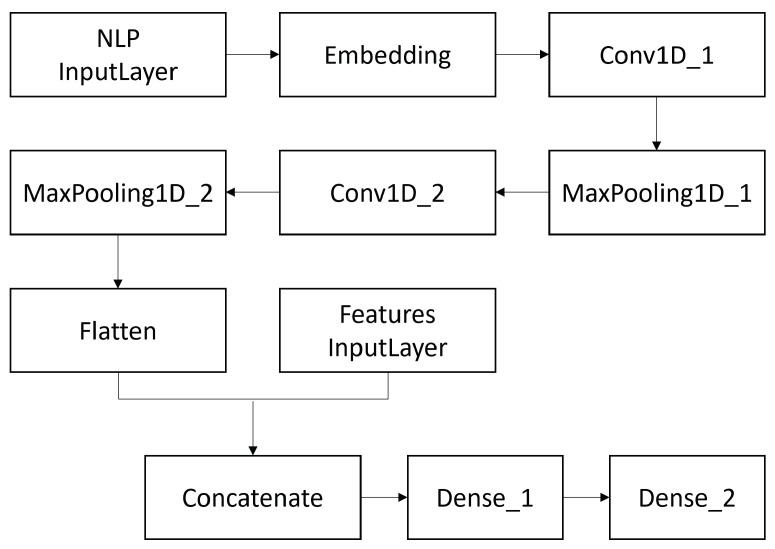
Architecture of the Convolutional Neural Network.

A *Convolutional Neural Network* (CNN) classifier, whose architecture is illustrated in Figure 2, was implemented.

As it emerges from the figure, the network takes as input an *embedding layer* created using the pre-trained vectors from GloVe. These are inserted into a *Conv1D layer*, whose high-level purpose is to train a number of 128 neurons (which represents the dimensionality of the output space). This layer has a *kernel size* of 5. The output of the layer is taken as input by a *MaxPooling1D layer*, whose *kernel size* is set, again, to 5 (the choice of such parameters is consistent with those that are usually employed in such architectures in the context of text classification). The introduction of such a layer allows us to reduce the output complexity. An additional *Conv1D layer* and *MaxPooling1D layer* are then created according to the same settings in order to train features of a higher level. These layers are then followed by a *Flatten layer* whose output is processed within a dense layer consisting of 128 neurons. The output of such layer is concatenated to the features considered in the proposed approach and finally processed in the dense *output layer* formed by 2 neurons to generate the binary classification. From a technical point of view, the *Adam* optimizer [75] was employed, while the considered loss function was the *binary cross-entropy* function [76]. Another tuning operation concerned the choice of the *vector size* for the word embedding representation; in the current study, vector sizes of 50, 100, and 200 elements were chosen. Regarding the number of *epochs* considered, the *callbacks* were set to save the trained model at the epoch in which the loss on the test data was minimized and stop the training in case of no improvement in the next 5 epochs.

#### 3.3.2. Bidirectional Long-Short Term Memory

**Figure 3:**
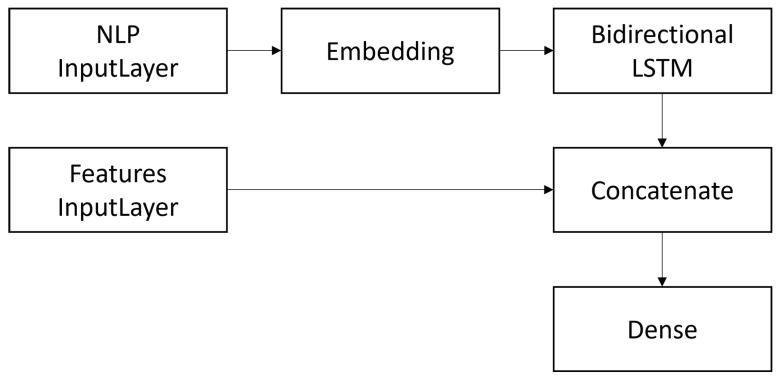
Architecture of the Bidirectional LSTM network.

A *Bidirectional Long-Short Term Memory* (Bi-LSTM) classifier was implemented according to the architecture shown in Figure 3.

It consists of an initial *embedding layer* containing GloVe’s word representations followed by a *Bidirectional LSTM* layer constituted by 128 neurons. To limit *overfitting*, a *dropout* of 0.3 and an *l2 regulator* were set. The output of the aforementioned layer is concatenated to the other features considered in this study, and then with a dense output layer consisting of 2 neurons to generate the binary classification. Additionally, in this case, the *Adam* optimizer was employed, and the loss function was the *binary cross-entropy* function. The same word embedding sizes were considered, i.e., vectors of 50, 100, and 200 elements. Regarding the number of *epochs*, *callbacks* were set as in the case of the CNN architecture.

## 4. Results

In this section, we present the results obtained by employing the health misinformation features illustrated in Section 3.2 in association with the binary classifiers detailed above. In particular, we introduce evaluation metrics and technical details, the effectiveness of the different classifiers with respect to different feature configurations, and the effectiveness of specific feature classes in health misinformation detection.

### 4.1. Evaluation Metrics and Technical Details

In the context of evaluating the effectiveness of the considered features and machine learning approaches in performing binary classification, genuine information (as labeled in the considered datasets) has been treated as the *positive class* and, conversely, not genuine information has been considered the *negative class*. As evaluation metrics, we considered the *Area Under the ROC Curve* (AUC) measure and the *f-measure* [77]. *Stratified* 5-*fold cross-validation* has been used to perform all assessments, by employing the *scikit-learn* library implementation [78]. Furthermore, to prevent issues related to possible *redundancy* and *multicollinearity*, the *Correlation-based Feature Selection* (CFS) approach proposed in [79] has been applied during cross-validation.

### 4.2. Global Evaluation Results

First, “classical” ML algorithms, such as *Gradient Boosting*, *Logistic Regression*, *Naïve Bayes*, and *Random Forests* are considered. Since textual representation features are based on three different representation models, the following three classification configurations are assessed:

- **ML(BoW-binary+all)**: ML algorithms in association with textual representation features (Bag-of-Words with binary weights) + all other features;
- **ML(BoW-TF-IDF+all)**: ML algorithms in association with textual representation features (Bag-of-Words with TF-IDF weights) + all other features;
- **ML(WE+all)**: ML algorithms in association with textual representation features (word embeddings) + all other features.

As for ML and the other binary classifiers considered in this paper, they are first evaluated against using baseline architectures and features as done in the literature [36,38,39,42,43,44,45]. In this case, they are denoted as:

- **Bi-LSTM(WE)**: Bidirectional Long-Short Term Memory classifier in association with only textual representation features (word embeddings);
- **CNN(WE)**: Convolutional Neural Network classifier in association with only textual representation features (word embeddings);
- **HPN**: Hierarchical Propagation Networks in association with the propagation-network features, as proposed in [42];
- **ML(LIWC)**: ML algorithms employed in association with the LIWC features proposed in [37].

Finally, CNN and Bi-LSTM classifiers are tested with respect to the use of word embedding features together with all the other features considered:

- **CNN(WE+all)**: Convolutional Neural Network classifier in association with textual representation features (word embeddings) + all other features;
- **Bi-LSTM(WE+all)**: Bidirectional Long-Short Term Memory classifier in association with textual representation features (word embeddings) + all other features.

In Table 4, Table 5 and Table 6, the values obtained as a result of the global evaluation are reported for each considered dataset. The best AUC and *f-measure* scores are shown in bold. The classification configurations are ordered according to their AUC scores. Result differences are statistically significant according to the *t*-test employed [80]. For ML classifiers, only the *best performance* obtained among the four considered algorithms is shown in the tables.

### 4.3. Feature Class Evaluation Results

This section illustrates the effectiveness of each class of features in health misinformation detection, except for textual representation features. In fact, as it emerges from Table 4, Table 5 and Table 6, and as further detailed in Section 5, textual representation features are of fundamental importance in all approaches considered, so we aim at assessing the discriminating power of the other feature classes. To do so, each distinct feature group is tested using the same “classical” ML algorithms, with the same default hyper-parameters of the *sklearn* library. Table 7 shows, for each dataset, the *best performance* obtained by each class of features in terms of AUCs. In the end, the best performing ML classifier for all datasets turned out to be the one based on *Random Forests*. From the table, it emerges that there is no class of features able to return, uniquely for each *dataset*, a higher AUC value than the others.

## 5. Discussion

Regarding the problem of identifying online health misinformation, through the features considered in this work and applied in the context of supervised machine learning, several considerations can be made about the results presented in the previous section.

### 5.1. Global Evaluation

As for the results of the *global evaluation*, we can affirm that, for all datasets considered, there is no superior *classifier-feature* configuration compared to all the others. However, we can state that, at the level of the distinct datasets:

- *CoAID*: the CNN(WE) and CNN(WE+all) configurations are superior on every metric compared to all other configurations. ML(WE+all) is superior to ML(BoW) on both AUC and *f-measure*;
- *ReCOVery*: the ML(WE+all) configuration is superior in terms of AUC and *f-measure* to ML(BoW), and it is comparable to CNN(WE) in terms of both metrics;
- *FakeHealth* (*Release*): ML configurations (with both WE and BoW representations together with all the other features) are superior in terms of both AUC and *f-measure* to all other configurations;
- *FakeHealth* (*Story*): in terms of AUC, the ML(BoW-TF-IDF+all), CNN(WE), CNN(WE+all), and ML(LIWC) configurations turn out to be not superior to each other. ML(WE+all) and ML(LIWC) are both superior to all other configurations considering the *f-measure*. This is the only dataset for which ML(LIWC) has proven to be effective.

From these and other observations on each dataset and each configuration considered (see Table 4, Table 5 and Table 6) we can say that, in general, the inclusion of *all features* in the CNN classifiers does not seem to lead to improvements compared to the use of only textual representation features (word embeddings). There is some improvement, instead, in considering all the features together with the Bi-LSTM classifiers, which, however, have always a lower effectiveness than using the CNN- and ML-based configurations. Such solutions are therefore always—except for the *FakeHealth* (*Story*) dataset—significantly superior to the other configurations. On a statistical level, on the *FakeHealth* (*Release*) dataset ML > CNN, while on the *CoAID* dataset CNN > ML; on the remaining datasets, no statistically significant difference in results are observed. ML(LIWC) and HPN always have suboptimal performance, except in the *FakeHealth* (*Story*) dataset where ML(LIWC) is effective as the best classifiers. This aspect may be related to the peculiarity of the *FakeHealth* (*Story*) dataset of having generated the absolute largest number of social reactions (see Table 2), which are often highly polarized. In a case like this, the use of LIWC-based features, which counts words in psychologically meaningful categories, could be particularly effective. This aspect deserves a definite investigation in the future.

### 5.2. Feature Class Evaluation

Regarding the evaluation of the effectiveness of different *feature classes* (except for textual representation features) per dataset in detecting online health misinformation:

- *CoAID*: user-profile and propagation-network features are particularly effective to tackle the problem under consideration; conversely, textual features such as linguistic-emotional, linguistic-stylistic, and linguistic-medical features are less performing;
- *ReCOVery*: also, in this case, user-profile and propagation-network features are those most suitable for the problem at hand, even if linguistic-stylistic features also show good effectiveness;
- *FakeHealth* (*Release*): linguistic-stylistic features show the best performance, followed, respectively, by user-profile and linguistic-medical features;
- *FakeHealth* (*Story*): linguistic-medical and linguistic-emotional features are those presenting the best effectiveness.

These observations allow us to surmise that, for similar types of health-related content, the feature classes that are most effective in detecting health misinformation are similar, and may depend on some intrinsic characteristics of both the *topic* and the *style* (i.e., the linguistic register and vocabulary employed) of the content under consideration. In fact, both *ReCOVery* and *CoAID* are made up by content mainly in the form of news and claims related to COVID-19, whose genuineness is more effectively evaluated by the *propagation-network* and *user-profile features*. Such content is often intended for the general public, and in most cases uses vocabulary that is not too formal. On the contrary, *FakeHealth* (*Release*) and (*Story*) contain health-related content aimed at describing medical therapies, health treatments, surgeries, etc., which arguably uses a more complex linguistic register and a complex medical-scientific vocabulary. For such types of medical information, the *linguistic-stylistic* and *linguistic-medical features* are the ones best suited to the problem addressed by this article. As also illustrated above, the *FakeHealth* (*Story*) dataset is the one that generated the most reactions on Twitter, which is perhaps why the linguistic-emotional feature category is particularly effective in this particular case.

## 6. Conclusions and Future Research

In the context of online health misinformation detection, this article carried out an overview and a study on how to succeed in assessing the genuineness of health-related content by means of supervised learning in association with suitable health misinformation features. Despite the fact that several studies have been carried out to tackle the same issue in the context of “general” misinformation detection, and some recent studies have focused on the health domain, in this article we have investigated and evaluated some theoretical and technological aspects that still deserve to be considered, especially regarding the specific characteristics of health information and the recent availability of some publicly available datasets, to avoid the disease-specificity of some recently proposed approaches.

The obtained results show that deep learning solutions are effective when using word embedding features obtained from appropriate training on medical vocabulary, without the need to use other types of features. However, when “classical” machine learning classifiers are used, the importance of considering other types of features increases. In particular, it has been observed that for health-related content that uses a more informal language, propagation-network features and user-profile features are particularly effective; when dealing with more formal medical content, linguistic-stylistic and linguistic-medical features are the most suitable. In particular, when content generates a high volume of social reactions, linguistic-emotional features can also make an important contribution.

In spite of these interesting findings, this work needs to be followed by further investigations, which primarily concern the type of data taken into consideration and the respective labeling methods. In fact, it is essential to remember that datasets have only recently been developed for the analysis of the genuineness of medical information; these collections have been generated in a heterogeneous way, without common guidelines, applying different labeling processes of the information, which potentially refer to concepts of genuineness that are not always totally superimposable. This can have an effect on the quality of the results obtained in this work. Furthermore, another aspect of in-depth analysis concerns the effectiveness of particular classes of features with respect to both the use of particular classifiers and with respect to specific types of content related to health. In particular, given the observation of the effectiveness of textual representation features (particularly word embedding features) in association with CNNs, we estimate that it will be of great importance to further test semantic and possibly context-aware representation models for health misinformation detection, such as BERT appropriately pre-trained on medical terms, or other transformer-based models.

## Figures and Tables

**Table 1:** Dimensionality of the original datasets.

Data	CoAID	ReCOVery	FakeHealth (Release)	FakeHealth (Story)
Textual contents	3555	2029	606	1690
Tweet IDs	151,964	140,820	47,338	384,073
Retweet IDs	-	-	16,959	92,758
Reply IDs	122,150	-	1575	20,644

**Table 2:** Dimensionality of the gathered and cleaned datasets.

Data	CoAID	ReCOVery	FakeHealth (Release)	FakeHealth (Story)
Textual contents	1820	1910	594	1498
Tweet IDs	74,722	42,153	44,547	315,709
Retweet IDs	65,464	43,024	16,070	99,971
Replies IDs	29,969	-	1253	14,472
User IDs	164,891	58,495	28,893	206,798

**Table 3:** List of the linguistic-stylistic features considered.

Features	Examples/Explanations
Strong modals	might, could, can, would, may
Weak modals	should, ought, need, shall, will
Conditionals	if
Negations	no, not, neither, nor, never
Conclusive conjunctions	therefore, thus, furthermore
Subordinating conjunctions	until, despite, in spite, though
Following conjunctions	but, however, otherwise, yet
Definite determiners	the, this, that, those, these
Personal pronouns	I, you
First person	I, we, me, my, mine, us, our
Second person	you, your, yours
Third person	he, she, him, her, his, it, its
Question particles	why, what, when, which, who
Adjectives	correct, extreme, long, visible
Adverbs	maybe, about, probably, much
Proper nouns	names of places, things, etc.
Other nouns	other nouns
To be form	be, am, is are, was, were, been
To have form	have, has, had, having
Past tense verb	past tense verb
Gerund	gerund
Participle verb	past or present participle verb
Superlatives	superlative adjectives or adverbs
Exclamation	exclamation mark
Other	other terms

**Table 4:** Global evaluation results for the CoAID dataset.

Dataset	Classifier	AUC	f-Measure
*CoAID*	CNN(WE)	**0.973**	**0.953**
	CNN(WE+all)	0.962	0.943
	ML(WE+all)	0.925	0.914
	ML(BoW-TF-IDF+all)	0.898	0.865
	ML(BoW-binary+all)	0.892	0.863
	Bi-LSTM(WE+all)	0.849	0.859
	Bi-LSTM(WE)	0.848	0.857
	HPN	0.844	0.858
	ML(LIWC)	0.669	0.789

**Table 5:** Global evaluation results for the ReCOVery dataset.

Dataset	Classifier	AUC	f-Measure
*ReCOVery*	ML(WE+all)	**0.921**	0.848
	ML(BoW-TF-IDF+all)	0.915	0.771
	CNN(WE)	0.913	**0.850**
	ML(BoW-binary+all)	0.903	0.709
	CNN(WE+all)	0.896	0.828
	ML(LIWC)	0.817	0.743
	Bi-LSTM(WE+all)	0.741	0.655
	Bi-LSTM(WE)	0.734	0.673
	HPN	0.716	0.694

**Table 6:** Global evaluation results for the FakeHealth dataset.

Dataset	Classifier	AUC	f-Measure
*FakeHealth* (*Release*)	ML(BoW-TF-IDF+all)	**0.693**	0.653
	ML(WE+all)	0.687	**0.658**
	ML(BoW-binary+all)	0.675	0.641
	CNN(WE)	0.661	0.602
	CNN(WE+all)	0.645	0.597
	ML(LIWC)	0.608	0.598
	Bi-LSTM(WE)	0.583	0.574
	Bi-LSTM(WE+all)	0.563	0.539
	HPN	0.581	0.593
*FakeHealth* (*Story*)	ML(BoW-TF-IDF+all)	**0.717**	0.627
	CNN(WE)	0.700	0.624
	CNN(WE+all)	0.698	0.655
	ML(LIWC)	0.694	0.704
	ML(BoW-binary+all)	0.679	0.609
	ML(WE+all)	0.657	**0.706**
	Bi-LSTM(WE+all)	0.656	0.602
	Bi-LSTM(WE)	0.654	0.602
	HPN	0.563	0.660

**Table 7:** Evaluation of effectiveness by feature class.

AUC	CoAID	ReCOVery	FakeHealth (Release)	FakeHealth (Story)
*Linguistic-emotional*	0.624	0.708	0.576	0.630
*Linguistic-stylistic*	0.601	0.774	**0.625**	0.532
*Linguistic-medical*	0.610	0.612	0.595	**0.633**
*Propagation-network*	0.729	**0.886**	0.525	0.548
*User-profile*	**0.847**	0.795	0.602	0.563

## Data Availability

The employed datasets, together with the code developed to extract health misinformation features and implement the classifiers, and more details about the considered features, are publicly accessible in the GitHub repository of the *Information and Knowledge Representation, Retrieval and Reasoning Laboratory* (IKR3 Lab), at the following link: https://github.com/ikr3-lab/health-misinformation/ (accessed on 3 February 2022).
